# A Crown and Bridge Case, 1859

**Published:** 1889-09

**Authors:** 


### A Crown and Bridge Case—1859.

At the recent meeting of the Michigan Dental Society held at Grand Rapids, we were put in possession of one of the oldest pieces of crown and bridge work, at least of modern times, that it has been our fortune to see. It was made by Dr. Day, of Grand Rapids, in 1859 for a Mr. ———, of Dexter, in the State of Michigan. It was worn and did good service for eight years, when the teeth to which it was attached became loose, and had to be removed.

It consists of a cap-crown for a second superior bicuspid, made of silver plate, and it appears in form just as the crowns that are now made. The first molar adjacent to this was absent and was replaced by a porcelain substitute which was attached to an arm or bridge which was soldered to the crown of the bicuspid and extended into a groove made for it in the anterior proximate surface of the second molar, and anchored by filling. This in construction is an exact likeness to many that are made at the present time. Dr. Day stated that since that time he has made quite a large number of pieces of crown and bridge-work. In the construction of this variety of work Dr. Day certainly antidates all those who have secured patents upon the work, and that too by many years.

Dr. Day exhibited other devices that showed native skill and inventive genius. Among these was a porcelain tooth crown, formed with a shoulder round the cervical part for the reception of a band which should embrace both it and the root, and for further attachment a staple of platinum wire is baked into the base of the tooth, into which an anchor loop is attached to be introduced into the root on which the crown is to be set, thus making a very firm attachment for an artificial crown.

